# Erratum: Correction of the Authors’ List and Affiliations 

**DOI:** 10.29252/beat-070419

**Published:** 2019-10

In the original investigation entitled “**Cervical Epidural Steroid Injection: Parasagittal versus Midline Approach in Patients with Unilateral Cervical Radicular Pain; A Randomized Clinical Trial.**” [1] which was published in April 2019 issue of the journal, one author name has been unintentionally omitted from the final draft and the affiliations were wrongly assigned. There is an error in the author list of this article, which should be corrected as follows: “Masoud Hashemi,^1^ Payman Dadkhah,^1^ Mehrdad Taheri,^1^ Ebrahim Golmakani,^1,2^ Kasra Dehghan,^1^ Rouhollah Valizadeh.^3^”. The affiliations are also corrected as follow: “^1^Pain Management Fellowship, Department of Anesthesiology, Anesthesiology Research Center, Shahid Beheshti University of Medical Sciences, Tehran, Iran; ^2^Department of Anesthesiology, Mashhad University of Medical Sciences, Mashhad, Iran; ^3^Department of Epidemiology, Student Research Committee, Iran University of Medical Sciences, Tehran, Iran”. The article was corrected online. 
